# Speech Sound Processing Deficits and Training-Induced Neural Plasticity in Rats with Dyslexia Gene Knockdown

**DOI:** 10.1371/journal.pone.0098439

**Published:** 2014-05-28

**Authors:** Tracy M. Centanni, Fuyi Chen, Anne M. Booker, Crystal T. Engineer, Andrew M. Sloan, Robert L. Rennaker, Joseph J. LoTurco, Michael P. Kilgard

**Affiliations:** 1 School of Behavioral and Brain Sciences, University of Texas at Dallas, Richardson, Texas, United States of America; 2 Physiology and Neurobiology, University of Connecticut, Storrs, Connecticut, United States of America; Rutgers University, United States of America

## Abstract

*In utero* RNAi of the dyslexia-associated gene *Kiaa0319* in rats (KIA-) degrades cortical responses to speech sounds and increases trial-by-trial variability in onset latency. We tested the hypothesis that KIA- rats would be impaired at speech sound discrimination. KIA- rats needed twice as much training in quiet conditions to perform at control levels and remained impaired at several speech tasks. Focused training using truncated speech sounds was able to normalize speech discrimination in quiet and background noise conditions. Training also normalized trial-by-trial neural variability and temporal phase locking. Cortical activity from speech trained KIA- rats was sufficient to accurately discriminate between similar consonant sounds. These results provide the first direct evidence that assumed reduced expression of the dyslexia-associated gene *KIAA0319* can cause phoneme processing impairments similar to those seen in dyslexia and that intensive behavioral therapy can eliminate these impairments.

## Introduction

Dyslexia is the most common developmental language disorder and affects approximately 7% of the population [1], [2]. Individuals with this disorder have normal nonverbal intelligence, but score more than a standard deviation below their peers on reading tests [1], [3], [4]. Children and adults with dyslexia typically have deficits in phoneme perception and manipulation [5]–[8]. Even before learning to read, neural activation during phonological processing is impaired in young children at risk for dyslexia [9], which suggests that the neural abnormalities responsible for dyslexia are present from birth and do not reflect experience-dependent processes [10].

Dyslexia is highly heritable and at least four candidate-dyslexia genes have been identified (*KIAA0319, DYX1C1, DCDC2 and ROBO1*) [11]–[14]. *KIAA0319* is the most consistently associated gene and the link between *KIAA0319* and dyslexia has been replicated in many independent studies [12], [15]–[20]. The observation that variants in *KIAA0319* impair speech evoked cortical activity [21] and cause poor speech perception and reading ability [19], [22] is consistent with the earlier hypothesis that phonological processing is a core deficit in dyslexia [23]–[30]. We have previously shown that *in utero* RNA interference (RNAi) of the rat homolog of this gene (*Kiaa0319*) is sufficient to impair neural processing of speech sounds by elevating trial-by-trial variability in the timing of neural responses [31]. *Kiaa0319* RNAi also duplicates corpus callosum abnormalities in dyslexia [23], [32], [33] without changing body weight or the volume of the cortex and hippocampus [34]. Based on the similarities between this gene's apparent function in rats and humans, we hypothesized that rats with reduced expression of *Kiaa0319* would exhibit impaired learning when trained to discriminate speech sounds.

Extensive auditory therapy has been used to treat millions of children with dyslexia. Many programs use acoustically modified speech stimuli to improve phoneme awareness [35]–[38]. Such training can cause changes in neural responses at multiple stages of the auditory pathway [37], [39]. These studies support the hypothesis that auditory training can induce therapeutic neural plasticity in dyslexia (but see [40]–[42]). The genotype of the participants in the intervention studies is unknown and it is not clear whether auditory training would be more or less effective in dyslexics with variants in particular dyslexia-associated genes.

In this study, we used an animal model of speech sound processing to evaluate the role of *Kiaa0319* in speech sound discrimination and training-induced plasticity. We trained control rats and rats with *in utero* RNAi of *Kiaa0319* (KIA- rats) on a variety of speech sound discrimination tasks designed to evaluate known deficits in human dyslexics, including problems with speech in noise, rapid speech sounds, and isolated phonemes. We recorded action potentials and local field potentials in primary (A1) and posterior auditory fields (PAF) after training and compared responses with untrained KIA- and control rats.

## Materials and Methods

### Ethics Statement

All transfection protocols were designed to minimize any animal suffering and were approved by the University of Connecticut Institutional Animal Care and Use Committee (Protocol # A12-027). All behavioral, surgical, and physiological procedures were designed to minimize any animal suffering and were approved by the University of Texas at Dallas Institutional Animal Care and Use Committee (Protocol # 99-06). Data presented in this study are available upon request.

### Animals

Subjects were Wistar rats, both male and female, that were young adults at the time of study. All rats used were subjected as embryos to *in utero* electroporation targeting lateral regions of neocortex that included the auditory cortex by methods described previously [16], [20], [31], [43]–[45]. In brief, pregnant Wistar rats were anesthetized on day 15 of pregnancy and the embryos were transfected with either an shRNA against *Kiaa0319* which can decrease the Kiaa0319 protein expression in cell culture (Figure S1) and can cause migration delay in neocortex in embryos. Note that this same shRNA vector and transfection procedure have been previously demonstrated to be effective at targeting and knocking down Kiaa0319 protein translation in rats [46]. Control transfection animals received a scrambled sequence control of *Kiaa0319* shRNA, also previously used, that contained 6 bases in the sequence scrambled to render the shRNA inactive in terms of reducing *Kiaa0319* expression [31]. *Kiaa0319* shRNA and scrambled shRNA constructs were injected at a concentration of 1.0 µg/µL. pB-GFP was co-transfected with the effective shRNA construct, and pB-mRFP was co-transfected with the scrambled *Kiaa0319* shRNA control construct to identify the experimental condition in post experimental histological analysis.

### Analysis of Transfection Efficacy

The experimental status of the subject remained blind to experimenters throughout the behavior and electrophysiological portions of the study. Following data collection, each subject was perfused transcardially with 250 mL of 0.1 M PB solution with 0.02% heparin, followed by 500 mL of 4% formalin solution in 0.1 M PB. Sections were taken at 80 µm intervals and analyzed under a confocal microscope (Zeiss) to identify the experimental status of each subject (green florescent protein marked experimental subjects and red florescent protein marked control littermates). The number of fluorescent cells was counted in a 1 mm^2^ area of layer 2/3 of primary auditory cortex bilaterally. These layers were used as this is where the highest concentration of transfected neurons were located. This number was then divided by the estimated number of cells in matching primary auditory cortex regions of this size to calculate a percentage of affected cells. The estimated number of neurons was calculated by manually counting the total number of all cells in areas of layer 2/3 auditory cortex that matched the regions used in the transfected-neuron analysis and then estimating neuron density over the 1 mm^2^ area.

### Behavioral Training

We trained 26 rats to discriminate a target speech sound (/dad/) in 4 different contexts. Of these rats, 16 received RNAi of *Kiaa0319* (KIA-) and 10 received scrambled RNAi and served as controls. The behavior tasks we tested are described in detail elsewhere [47]–[50]. Briefly, rats were trained to respond to a target sound /dad/ using either a lever press or withdrawal from an infra-red nose poke. Once rats understood the mechanism of response (either a lever press or a withdrawal from the nose poke), rats were trained to wait for the presentation of a target sound prior to making a response. Once rats reached a d' of > = 1.5 for 10 sessions, they were moved on to a minimum of 20 sessions of each of four discrimination tasks [51].

The isolated speech task consisted of a go-no go paradigm in which rats were trained to press a lever in response to the target sound and to reject each of seven distractors: /dad/ versus /bad/, /gad/, /sad/, /tad/, /dud/, /deed/, /dood/ [47]. Rats were rewarded with a food pellet if they pressed within 3 second (s) of the target and punished with a 6 s time out if they false alarmed. The speech in noise task used the same stimuli with the addition of four levels (0, 48, 60, 72 dB SPL) of continuous speech-shaped noise [52]. In this task, trials were presented in blocks of gradually increasing or decreasing noise to allow rats to adjust to the noise. The truncated speech task was identical to the go-no go discrimination task except that only the first 40 ms of each speech sound was presented [49]. The rapid speech task presented a random sequence of distractor sounds (/bad/, /gad/, /sad/, /tad/), with the target sound (/dad/) inserted randomly between 2–7 s from the start of the trial. Sounds were only delivered while the rat's nose was inside an infra-red nose poke [50]. Rats were rewarded with a food pellet if they removed their nose within 500 ms of the target and punished with a 6 s time out if they false alarmed. Percent correct is reported as the average hits-false alarms for each task.

### Acute neural recordings

Following the approximately 4 months of training needed to complete all 4 tasks; rats were anesthetized with dilute pentobarbital and mapped. The techniques used for acute recordings are described in detail elsewhere [31], [52]–[55]. In brief, animals were anesthetized with pentobarbital (50 mg kg^−1^) and multi-unit recordings were acquired at cortical layer 4/5 (∼600–800 µm) using four Parylene-coated tungsten microelectrodes (1–2 MΩ). We used previously recorded multiunit responses from 11 experimentally naïve rats to evaluate the effect of training on neural responses; data from 5 untrained KIA- rats and 6 naïve controls was previously published in Centanni et al. 2013a. Each experimental group (untrained, group 1 trained and group 2 trained) underwent neural recordings once for the purposes of this study. Untrained rats were recorded from as described previously [31], while group 1 and group 2 rats were recorded from following their 4 months of behavioral training.

At each site, we presented a tuning curve consisting of 90 frequencies (1–47 kHz) at 16 intensities (0–75 dB SPL) to determine the characteristic frequency (CF) of each site, trains of six broadband noise bursts (presented 4, 7, 10 and 12.5 Hz) to evaluate following ability of A1 neurons, and the speech stimuli used in our behavior tasks [31], [47], [49], [52], [55]. Speech sounds were recorded in a double-walled, soundproof booth and were spoken by a female, native English speaker. The spectral envelope was shifted up in frequency by a factor of two using the STRAIGHT vocoder [56] to better accommodate the rat hearing range (Figure S3).

### Analysis of neural recordings

Though behavior did not differ between groups, we analyzed tuning curves for each group to see if the training order caused differences in the neural responses. To define A1 and PAF sites, multi-unit recording sites were manually analyzed to select the CF of each site, as well as to obtain bandwidth, latency, peak firing and end of peak response information. A1 sites were defined as having a short onset latency (∼15 ms), narrow bandwidths, and tonotopic organization so that CF increased in a posterior to anterior direction. PAF sites were defined based on their long onset latency (>30 ms), broad bandwidths, and poorly defined tonotopic organization. This method of characterizing auditory fields has been previously validated [55], [57]–[62]. Sites that were not from A1 or PAF were not analyzed further.

We trained half of the rats using one task order (group 1 rats: isolated speech, speech in noise, rapid speech, truncated speech) and the other half using a second task order (group 2 rats: truncated speech, isolated speech, speech in noise, rapid speech). This change in order was to determine if KIA- rats would benefit from specific truncated speech training, as is often used in humans with dyslexia. Control A1 and PAF sites were less variable and more accurate at the consonant classifier task following the second task order compared to control A1 and PAF sites after the first task order (unpaired t-tests for variability and classifier performance; p<0.01), while there was no difference in KIA- sites across training task orders. Since there were only minor differences across groups in the neural responses following training, neural activity from both groups was combined for analysis.

In response to broad band click trains, normalized spike rate (number of spikes evoked by bursts 2–6, normalized by the number of spikes to the first burst) and vector strength (VS) were calculated. VS quantifies the degree of synchronization between action potentials and repeated sounds. Mean VS is calculated with the formula:
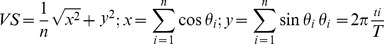
where n  =  total number of action potentials, t_i_ is the time of occurrence of the i'th action potential, and T is the inter-stimulus interval. Perfect synchronization would result in a value of one, whereas no synchronization would result in a value of zero.

We have previously shown that neural responses in KIA- rats are poor predictors of stimulus identity, while responses in control animals are good predictors of stimulus identity. To test whether training can improve KIA- neural responses, single trial response patterns to each of the isolated speech sounds were compared using a well-documented nearest neighbor classifier [31], [47], [48], [52], [63]–[65]. We used Euclidean distance to compare single trial activity to the average activity (PSTH) evoked by 19 repeats each of two different stimuli. For consonants, activity was binned using 1 ms temporal precision over a 40 ms window to encompass the spike timing precision present in the initial consonant [46], [48], [52], [54], while vowel activity was binned across a single 400 ms window so that only spike count information was preserved [47], [52]. The classifier then compared the response of each single trial with the average activity template (PSTH) of each of the speech stimuli presented. The current trial being considered was not included in the PSTH to avoid artifact. The classifier attempted to identify the stimulus that evoked the current single trial activity pattern by selecting the template that was most similar to the single trial in units of Euclidean distance. ED was calculated using the formula:
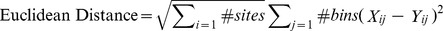
where n_sites_ is each recording site and n_bins_ is each of 40 one-millisecond bins being compared between activity evoked by speech sound X versus speech sound Y. For vowel sounds, the classifier counted the number of action potentials over 400 ms from a single trial and compared the value to the average response to each of the sounds [47], [52], [64]. We used two tailed t-tests for all pairwise comparisons of the accuracy of both classifiers and for comparison of basic neural firing properties across experimental groups. One-tailed t-tests were used to evaluate behavioral ability, as our previous data suggested that KIA- animals would have impairment on speech discrimination tasks [31]. 1-way ANOVA was used to compare vector strength across groups. Bonferroni correction was used to correct for multiple comparisons.

## Results

### *In utero* RNAi of *Kiaa0319* impairs speech sound discrimination

KIA- rats (N = 16) learned to detect a target speech sound as quickly as controls (N = 10). Both groups took approximately 8 days to reach the criterion of 10 training sessions with a d' above 1.5 (Controls: 8.4±0.3 days vs. KIA-: 9.6±0.6 days; p = 0.17; Figure S2A). Since ADHD and dyslexia have high comorbidity in humans, we evaluated response latency across groups to ensure that RNAi of *Kiaa0319* did not also cause hyperactivity. Significantly shorter response time has been linked to the presence of ADHD in both humans [66] and in rat models [67], [68]. Though on the first day of training, KIA- animals responded significantly faster, the groups were not significantly different on any other training day (Figure S2B). These results suggest that the assumed *in utero* knockdown of *Kiaa0319* does not cause significant hyperactivity, impulsivity, motor problems, or difficulty hearing speech sounds.

**Figure 1:**
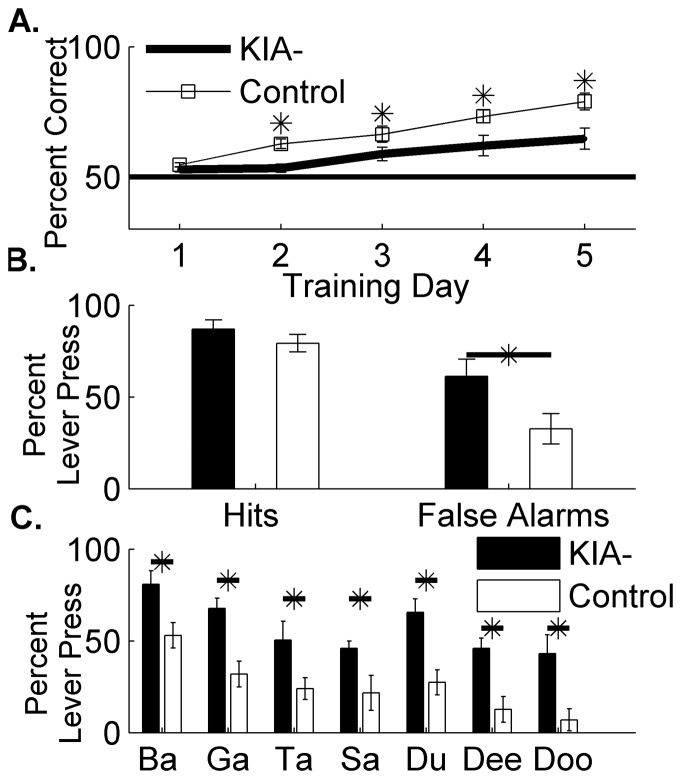
Rats with *in utero* RNAi of *Kiaa0319* are impaired at speech discrimination tasks. **A**. Performance of KIA- and control rats on the first 5 days of full length speech training. KIA- rats were significantly worse than control rats on the full speech discrimination task on 4 of the days (* = p<0.01). **B**. On day 5 of testing, KIA- rats hit to the target sound dad at the same rate as control rats (unpaired t-test; p = 0.33), but false alarmed to the distractor sounds significantly more than control rats (* = p = 0.04). **C**. Break down of lever press rates on day 5 of testing to each of the distractor sounds. KIA- rats responded to every sound significantly more than control rats (unpaired t-tests, * = p<0.01).

Five KIA- rats and five control rats next learned a speech discrimination task in which they were required to press the lever to the target sound /dad/ and withhold pressing to the distractor sounds (/bad/, /gad/, /sad/, /tad/, /dud/, /deed/, /dood/; Figure S3). On the first day, controls and KIA- rats hit to all sounds and performed at chance (56.3±2.3% correct by controls vs. 51.1±1.1% correct by KIA- compared to 50% chance; p = 0.11 and p = 0.34 respectively; Figure 1A). On each of the next 4 days of testing, KIA- rats were significantly worse than controls at performing this task (last day performance was 64.7±4.0% vs. 78.9±3.3% correct by control rats; p<0.01; Figure 1A). KIA- rats false alarmed to distractor sounds almost twice as often as control rats (61.3±9.3% false alarms by KIA- rats vs. 32.7±8.2% false alarms by control rats; p = 0.04, Figure 1B&C).

### Rats with *in utero* RNAi of *Kiaa0319* remain impaired in adverse listening conditions

**Figure 2:**
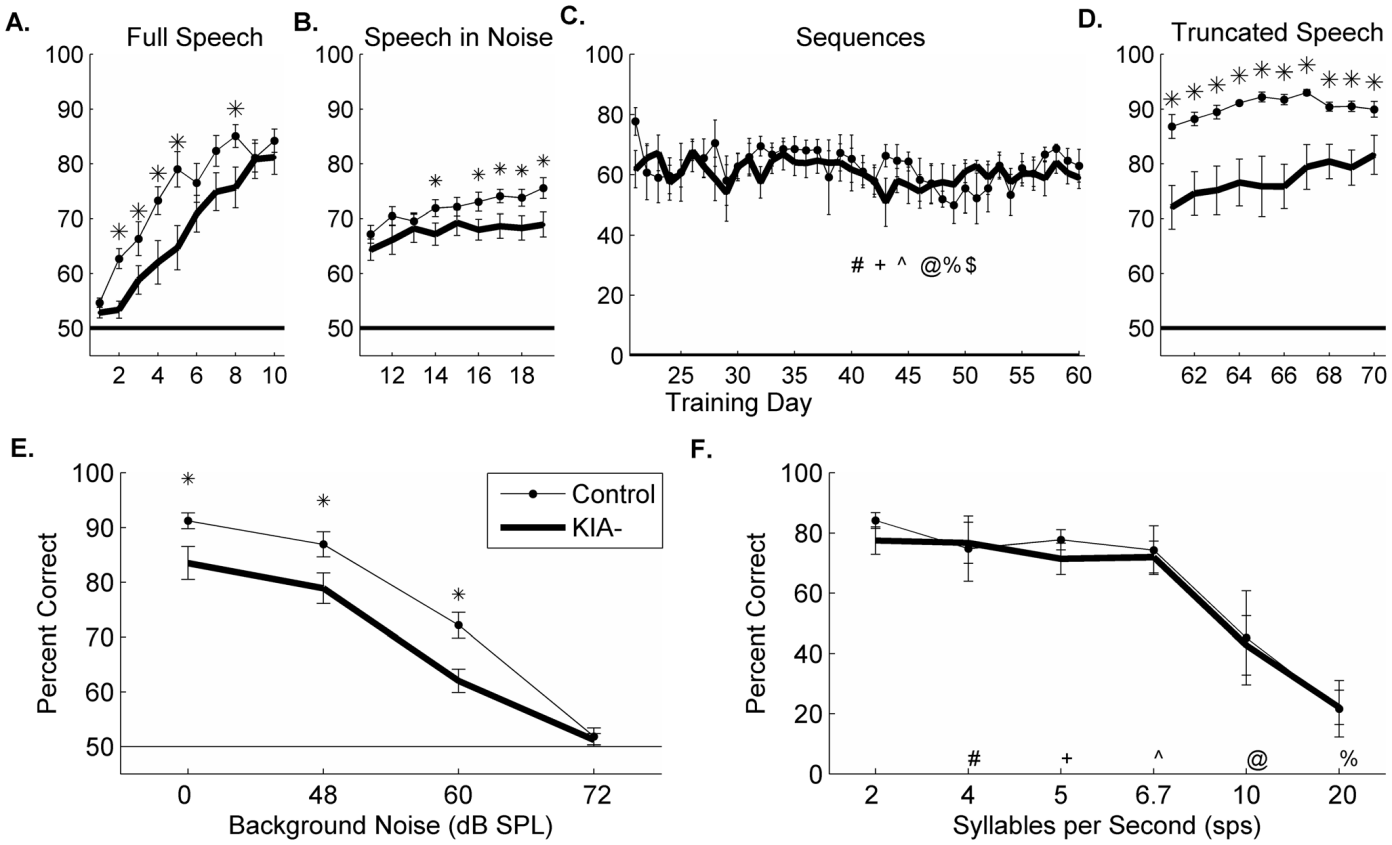
Extensive speech discrimination training can improve on clear speech tasks. Horizontal lines in each panel represent chance performance for that task. **A**. Timeline of performance on the full length speech task. After an additional week of training, 8 KIA- rats were able to perform the full speech task at the same level as 5 control rats (unpaired t-test, p = 0.24). **B**. Timeline of performance on speech in noise task. KIA- rats remained significantly below control levels at the end of training (* = p<0.05). **C**. Timeline of performance on sequence task. There were no significant differences between control and KIA- rats during this 40 day training period. Symbols correspond to the first day of training at each new stage (see panel F for symbol key). **D**. Timeline of performance on truncated speech task. KIA- rats were significantly impaired at this task compared to controls until the final day of training (* = p<0.01). **E**. Last day performance of rats on the speech in noise task. (* = p<0.01). **F**. Last day performance of rats on the sequence task. There were no significant differences between control and KIA- rats at any presentation rate tested (2 sps, p = 0.45; 4 sps, p = 0.68; 5 sps, p = 0.27; 6.67 sps, p = 0.65; 10 sps, p = 0.99; 20 sps, p = 0.74).

**Figure 3:**
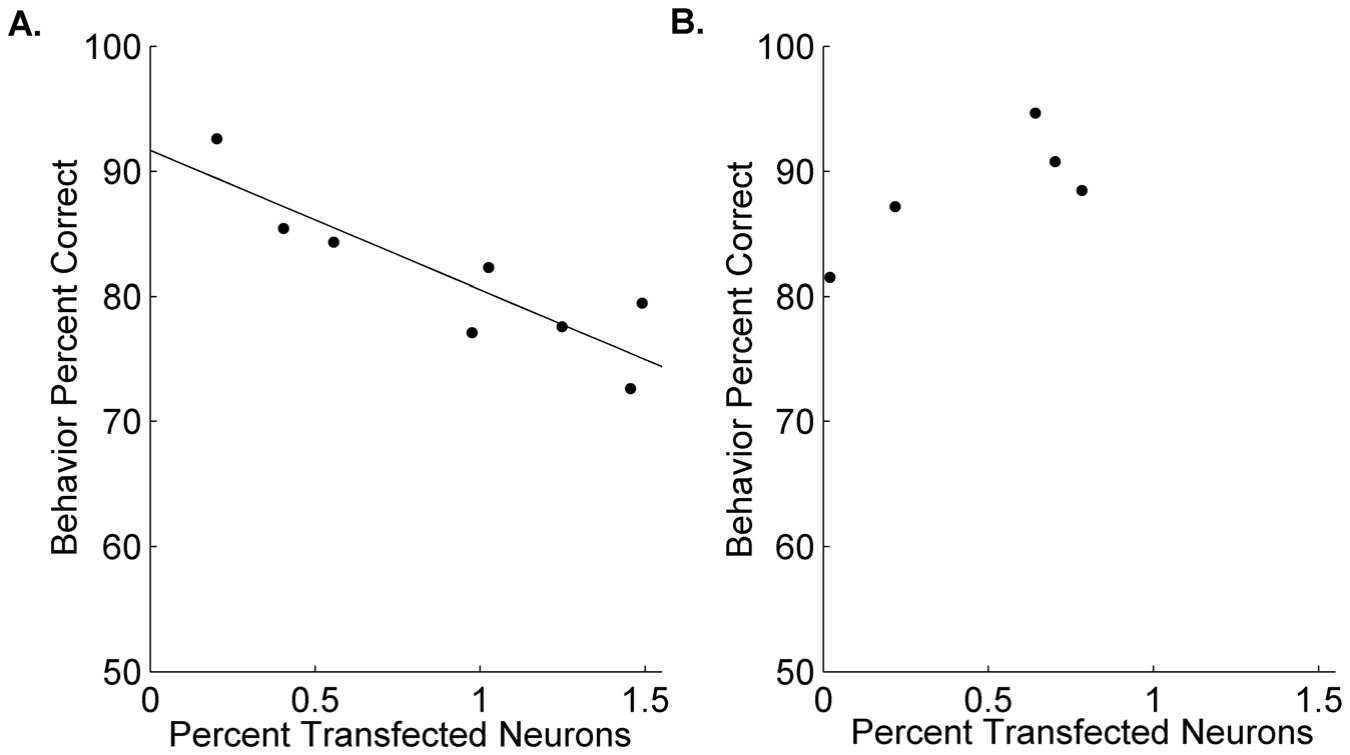
Percentage of transfected neurons predicts behavioral aptitude in KIA- rats. Correlation line denotes significance. **A**. The percentage of layer 2/3 pyramidal neurons affected by the transfection was calculated in A1 bilaterally. In KIA- rats, a higher percentage of transfected neurons was strongly correlated with impaired behavioral performance on the last day of full speech training (R = −0.66, p<0.01). **B**. The percentage of transfected neurons in control animals was not correlated with performance (R = 0.88, p = 0.12).

Dyslexia training programs often focus on intensive practice on phonological processing tasks [35]–[38]. We hypothesized that KIA- rats would improve on the discrimination task with additional training. After 5 additional days, all KIA- rats reached 80% correct on the full speech task (81.4±2.3% correct on the last day of training). KIA- rats took fifty percent longer to reach this criteria compared to controls (9.6±0.6 days of training vs. 6.2±0.6 days for control rats, p<0.01; Figure 2A). To test whether differences in RNAi transfection rate were responsible for differences in performance across rats, we compared the percent of transfected neurons with the last day performance on the full speech discrimination test. In rats with *in utero* RNAi of *Kiaa0319*, the percent of affected neurons was strongly negatively correlated with speech discrimination performance. Rats with a greater percentage of affected neurons were more impaired at the task than rats with fewer affected neurons (R = −0.66, p<0.01; Figure 3A). The percentage of transfected neurons in control rats was not correlated with behavioral performance (R =  0.88, p = 0.12; Figure 3B). The lack of correlation in our control rats suggests that the surgery itself did not cause the behavioral impairment, and that the deficit seen in KIA- rats was due to RNAi of the candidate-dyslexia gene *Kiaa0319*. Our results suggest that the degree of *in utero* transfection of this gene is related to each rats' aptitude for learning the full speech sound task. These results support our hypothesis that reduced expression of *KIAA0319* causes impaired phoneme discrimination.

People with dyslexia can often identify speech sounds accurately in quiet, but have a significant impairment on the same task in background noise [4], [69]–[71]. We hypothesized that KIA- rats would also have difficulty with speech discrimination in background noise. After two weeks of discrimination training in quiet (2 sessions/day, 5 days/week), rats were subsequently trained for two weeks on a speech in noise task. The same target and distractors from the full length task were used (presented at 60 dB SPL) and were presented in continuous speech-shaped background noise at one of four intensities (0, 48, 60, or 72 dB SPL) [52].

Control rats were able to improve over the course of 10 training days (last day performance and paired t-test of last day performance vs. first day; 0 dB 91.2±1.5% p<0.01; 48 dB 86.9±2.3% p<0.01; 60 dB 72.2±2.3% p<0.01; 72 dB 51.9±1.5% p = 0.38; Figure 2B&E). The inability to improve on the loudest intensity noise mimics previous chance level performance at this noise level using control rats [52]. KIA- rats were also able to significantly improve by the last day of training (last day performance and paired t-tests vs. first day performance; 0 dB 83.5±3.0% p<0.01; 48 dB 78.9±2.8% p<0.01; 60 dB 62.0±2.1% p = 0.11; 72 dB 51.2±1.2% p = 0.96), but remained significantly worse than control rats in both quiet and noise (p<0.01; Figure 2B&E). Rats with the knockdown had significantly different performance overall (2-Way ANOVA; F(1,116)  = 14.6, p = 0.0008), which did not change significantly over time (F(2,116)  = 1.09, p = 0.35). There was no significant interaction between experimental status and time (F(8,116)  = 0.80), which is not surprising since both controls and KIA- rats were able to significantly improve over the course of training. This result suggests that although KIA- rats are able to improve with training, they remain significantly worse than control rats at speech discrimination in a variety of contexts.

As shown previously, the auditory cortex of KIA- rats is significantly worse at following repetitive stimuli compared to controls [31]. We next trained rats on a speech discrimination task made difficult by high repetition rate [72]. A target speech sound (/dad/) was inserted into a random string of distractor speech sounds, and rats were trained to respond when the target sound was presented. Since this task used an infrared nose poke instead of a lever, training time was extended to allow the rats to learn the new response mechanism (see Materials and Methods). At each training stage (marked by symbols in Figure 2C&F), rats were introduced to faster presentation rates (2, 4, 5, 6.67, 10 and 20 syllables per second; sps). KIA- and control rats performed the task equally well during these stages (Figure 2C). During the last 10 days of training, rats were trained on all 6 presentation rates within a single session (in random blocks of 20 trials per presentation rate). Control and KIA- rats were not different in their accuracy on any of the presentation rates (Figure 2F). These results suggest that either KIA- rats do not have a behavioral consequence of their temporal processing deficit or that the neural deficit has been reversed by extensive training.

### KIA- rats are impaired at speech discrimination using only onset cues

Based on physiological recordings in A1 of KIA- rats, we hypothesized that rats may compensate for consonant identification deficits by using cues that occur outside the first 40 ms, such as duration or pitch [47], [55]. We truncated the speech sounds so that each contained only the initial 40 ms [49] and tested KIA- and control rat discrimination of these sounds for two weeks. On the first day of testing, control rats were significantly better than KIA- rats (89.7±0.6% correct by controls vs. 72.4±0.6% correct by KIA- rats; p<0.01; Figure 2D). This result suggests that KIA- rats had been performing the speech discrimination task using cues not present in the onset of the sound. KIA- rats remained significantly impaired (day 1–9, p<0.01; day 10, p = 0.08; Figure 2D), which suggests that KIA- rats have a persistent impairment at discriminating speech sound onsets. In spite of this persistent impairment compared to controls, KIA- rats were able to significantly improve over the course of the 10 days of training (paired t-test, p<0.01).

### KIA- rats can learn phoneme discrimination with extensive training

**Figure 4:**
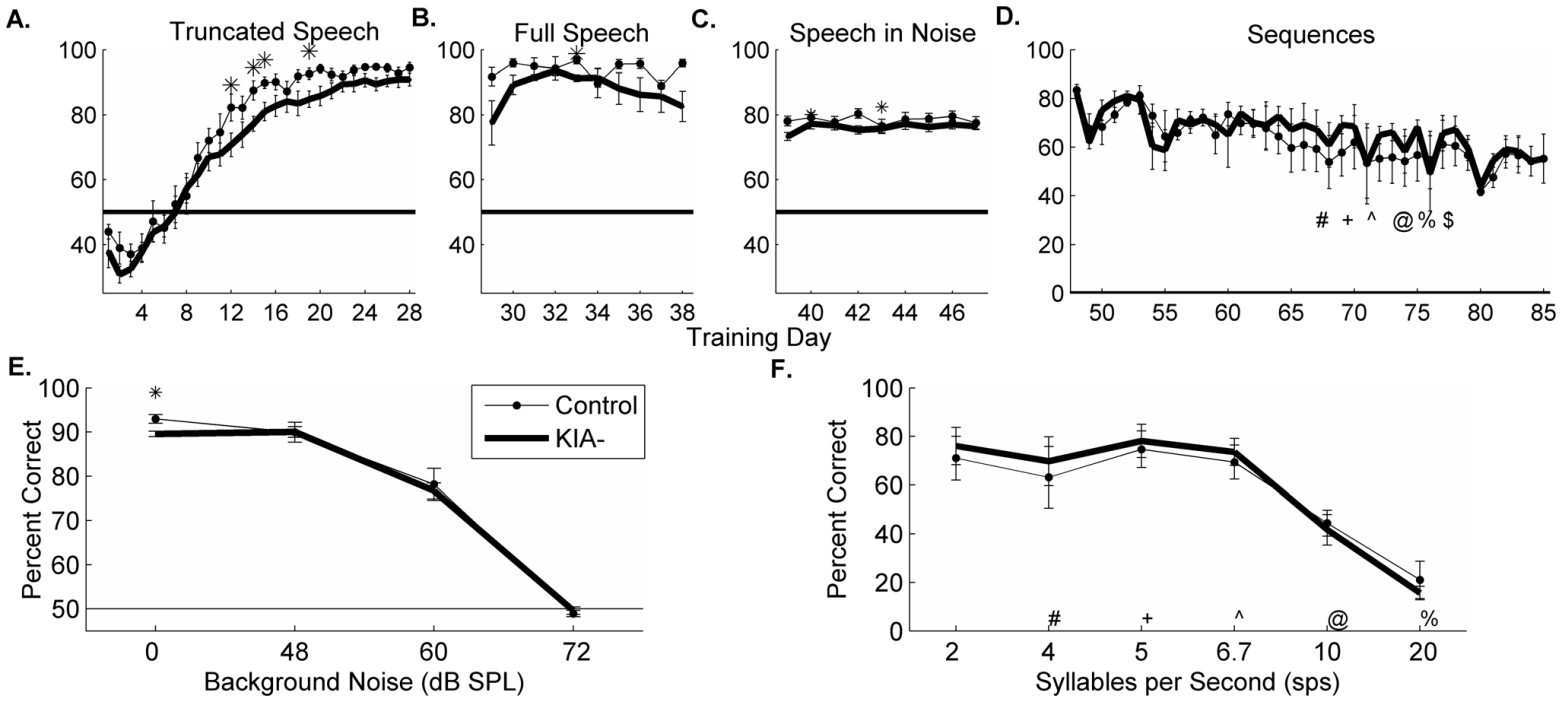
Extensive truncated speech training improves full speech and speech in noise performance in KIA- rats. Horizontal lines in each panel represent chance performance for that task. **A**. Timeline of group 2 rats' performance on truncated speech task. KIA- rats needed slightly longer to reach 80% correct compared to controls (unpaired 1-tailed t-test, p = 0.07). At the end of training, there was no significant difference in performance across groups (unpaired 1-tailed t-test, p = 0.11). **B**. Timeline of performance on full length speech task. **C**. Timeline of performance on the speech in noise task. **D**. Timeline of performance on the sequence task. Performance on this task falls slightly over time due to the addition of increased repetition rates. **E**. Last day of speech in noise performance by control and KIA- rats. **F**. Last day of sequence performance.

Auditory training in humans with dyslexia using truncated speech sounds generalizes to other speech tasks [37], [73]. We hypothesized that training KIA- rats on the truncated speech sounds from the beginning might improve performance on the other tasks since KIA- rats were able to improve on the truncated speech task over time. We trained a second group of 8 KIA- and 5 control rats on the truncated speech task for 28 days, which was the length of time needed for control rats to reach asymptotic performance [49]. This group of rats had no experimental training prior to beginning this set of tasks. All rats were first trained on shaping and detection as described above and then trained on discrimination using the truncated speech sounds. Both control and KIA- rats needed 7 days of training to perform above chance levels on this difficult task. KIA- rats required 33% longer to reach 80% correct, but survival curves were not statistically significant (16.3±2.1 vs. 12±1.2 days of training, survival analysis; log rank test, X^2^(1, N = 13) = 2.155, p = 0.14; Figure 4A). KIA- rats were significantly worse than controls on several training days, but performed as well as controls at the end of 28 days of training on the truncated speech task (90.8±1.9% vs. 94.5±1.6% correct, KIA- vs. controls respectively; p = 0.14). These results suggest that KIA- rats are able to learn to discriminate truncated speech sounds when the task is introduced early in training.

We then tested these rats on the other tasks to determine whether training on isolated phonemes would generalize to full length speech sounds. KIA- rats were able to perform the full speech task as well as control rats (average percent correct in KIA- rats was 87.7±3.1% vs. 93.9±2.2% in controls, p = 0.37; Figure 4B). KIA- rats performed as well as controls on the speech in noise task, although they were slightly worse during the blocks without background noise (0 dB p<0.01; 48 dB p = 0.97; 60 dB p = 0.71; 72 dB p = 0.53; Figure 4C&E). KIA- rats that received truncated speech training were not significantly different from controls during the speech sequence task (Figure 4D&F). These results suggest that truncated speech sound training benefits rats with *Kiaa0319* knockdown, especially in adverse learning conditions such as speech in noise.

### Extensive behavioral training restores neural firing patterns in KIA- auditory cortex

Since extensive behavioral training in normal rats and in human dyslexics can improve neural responses to speech and non-speech stimuli [37], [60], [74]–[76], we hypothesized that extensive speech training would improve neural responses in KIA- rats by reducing variability. We chose to evaluate the effect of training on neural responses in primary auditory cortex (A1) and posterior auditory field (PAF), because these fields have different response properties and training-induced plasticity may have affected these areas in unique ways [55], [58], [61], [75]. We compared the neural data from rats acquired after 4 months of behavioral training to the neural recordings in untrained rats previously described [31]. After training, A1 neurons in KIA- rats responded to tones 7 ms faster than in untrained KIA- rats (p<0.01; Figure S4A). After training, PAF neurons in KIA- rats responded to tones 15 ms faster than in untrained KIA- rats (p<0.01; Figure S4A). Training also reduced control responses by 5 ms in A1 and 10 ms in PAF (p<0.01 and p = 0.11, A1 and PAF respectively; Figure S4A).

**Figure 5:**
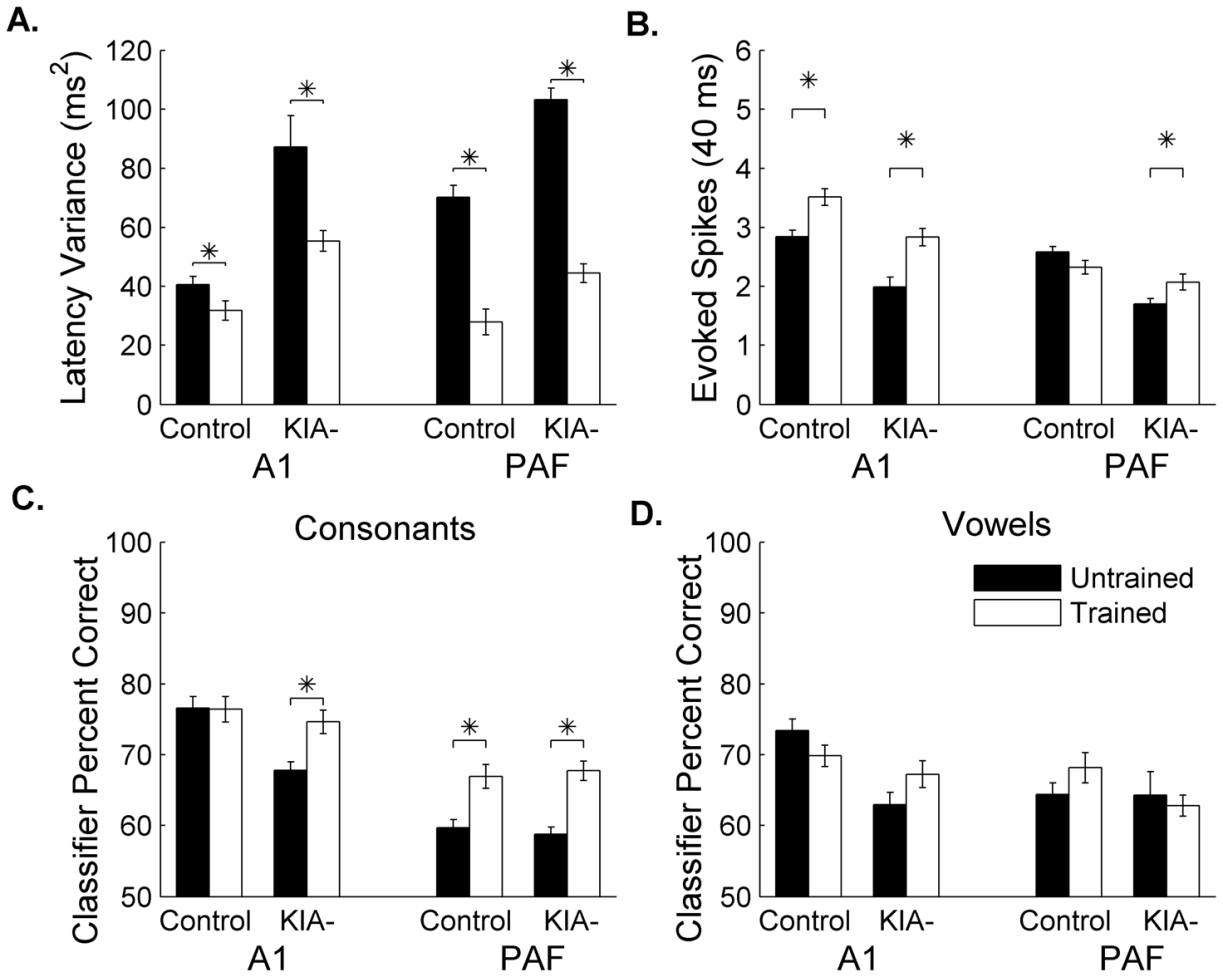
Extensive behavioral training improves reliability of neural firing and neural discrimination performance. **A**. Training significantly decreased the variability in onset latency in KIA- A1 (* = p<0.01) and KIA- PAF (* = p<0.01). Training also decreased variability in control A1 (* = p = 0.04) and control PAF (* = p<0.01). **B**. Training significantly increased the number of evoked spikes (* = p<0.04). **C**. Consonant classifier performance before and after training. (* = p<0.01). **D**. Vowel classifier performance before and after training.

**Figure 6:**
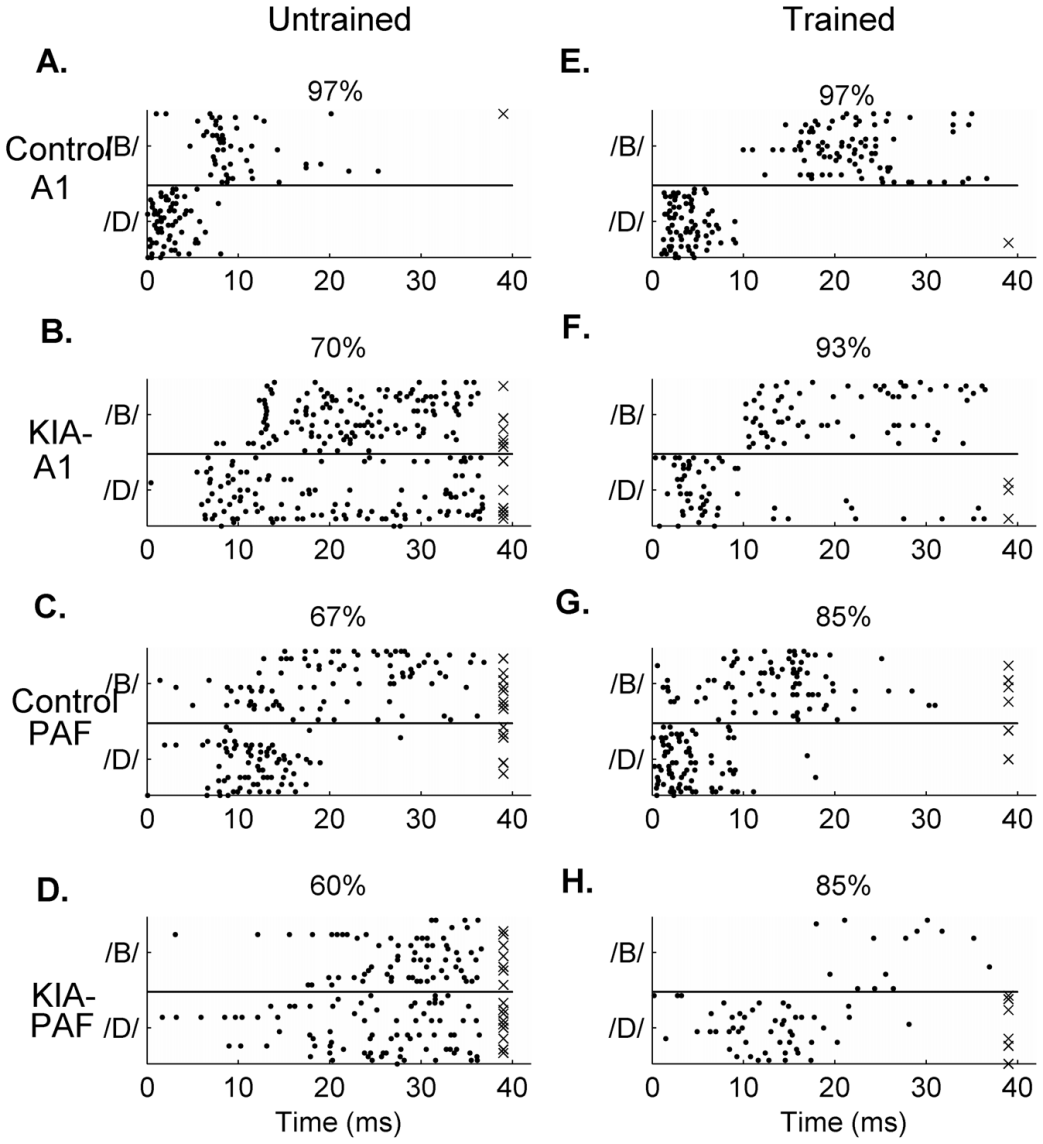
Training improves firing reliability in response to consonant speech sounds. Single site examples of neural responses to the consonant sounds /d/ and /b/ in every field before and after training. Classifier performance for each site is plotted on top of each panel, and trials which the classifier guessed incorrectly are marked by an ‘x’. **A–D**. Representative examples of single site responses to the consonants /b/ and /d/ in A1 and PAF of untrained control or KIA- rats. **E–F**. Representative examples of single site responses to the consonants /b/ and /d/ in A1 and PAF of control or KIA- rats after training was complete.

Speech training cut the trial-by-trial variability to speech-evoked responses in half by KIA- rats in A1 (87.3±10.51 ms^2^ in untrained vs. 55.3±3.5 ms^2^ after training; p<0.01; Figures 5A and 6F) and PAF (103.2±3.9 ms^2^ in untrained vs. 44.5±3.2 ms^2^ after training; p<0.01; Figures 5A and 6H). Training also decreased trial-by-trial variability in control A1 (40.6±2.7 ms^2^ in untrained vs. 31.8±3.3 ms^2^ after training; p = 0.04; Figures 5A and 6E) and control PAF (70.2±4.1 ms^2^ in untrained vs. 27.9±4.4 ms^2^ after training; p<0.01; Figures 5A and 6G). After training, the number of speech-evoked spikes in the onset response (the first 40 ms after stimulus presentation) significantly increased in control A1 and KIA- A1 and PAF sites (p<0.04; Figure 5B). Control PAF did not fire more spikes as a result of behavioral training (p = 0.07; Figure 5B).

We hypothesized that the improved reliability in trial-by-trial neural firing and increased response strength to sounds to would improve neural speech discrimination ability. Using a nearest-neighbor classifier, we compared performance using trained versus untrained neural recordings (see Materials and Methods). Training did not improve classifier performance using control A1 sites (consonant tasks: p = 0.94; vowel tasks: p = 0.33; Figures 5C&D and S5E&G). Training improved classifier performance on consonant tasks using PAF sites in control rats (p<0.01; Figures 5C and 6G) but did not improve vowel discrimination (p = 0.97; Figures 5D and S5G).

Training significantly improved classifier performance using A1 sites in KIA- rats on the consonant tasks (p<0.01; Figures 5C and 6F), but was less effective at improving vowel discrimination (p = 0.09; Figures 5D and S5F). Neural discrimination using A1 activity from trained KIA- rats was not significantly different from that achieved using trained control A1 sites (consonants p = 0.46; vowels p = 0.13). Training improved KIA- PAF sites' performance on neural consonant discrimination (p<0.01; Figures 5C and 6H) but not neural discrimination of vowels (p = 0.52; Figures 5D and S5H). These results suggest that extensive auditory training improves the ability of A1 and PAF in KIA- rats to accurately encode consonant speech sounds (which require temporal precision). We also noticed additional reduction in variability and additional improvement in the consonant classifier using control sites from the second group of rats as compared to the first, but did not notice any additional improvement in the second group of KIA- rats compared to KIA- rats in the first group (Figure S6). This result may suggest that there is a ceiling to the amount of training-induced neural plasticity in a brain with assumed *in utero* knockdown of *Kiaa0319* as compared to a control brain.

### Extensive behavioral training improves phase-locking in KIA- rats

**Figure 7:**
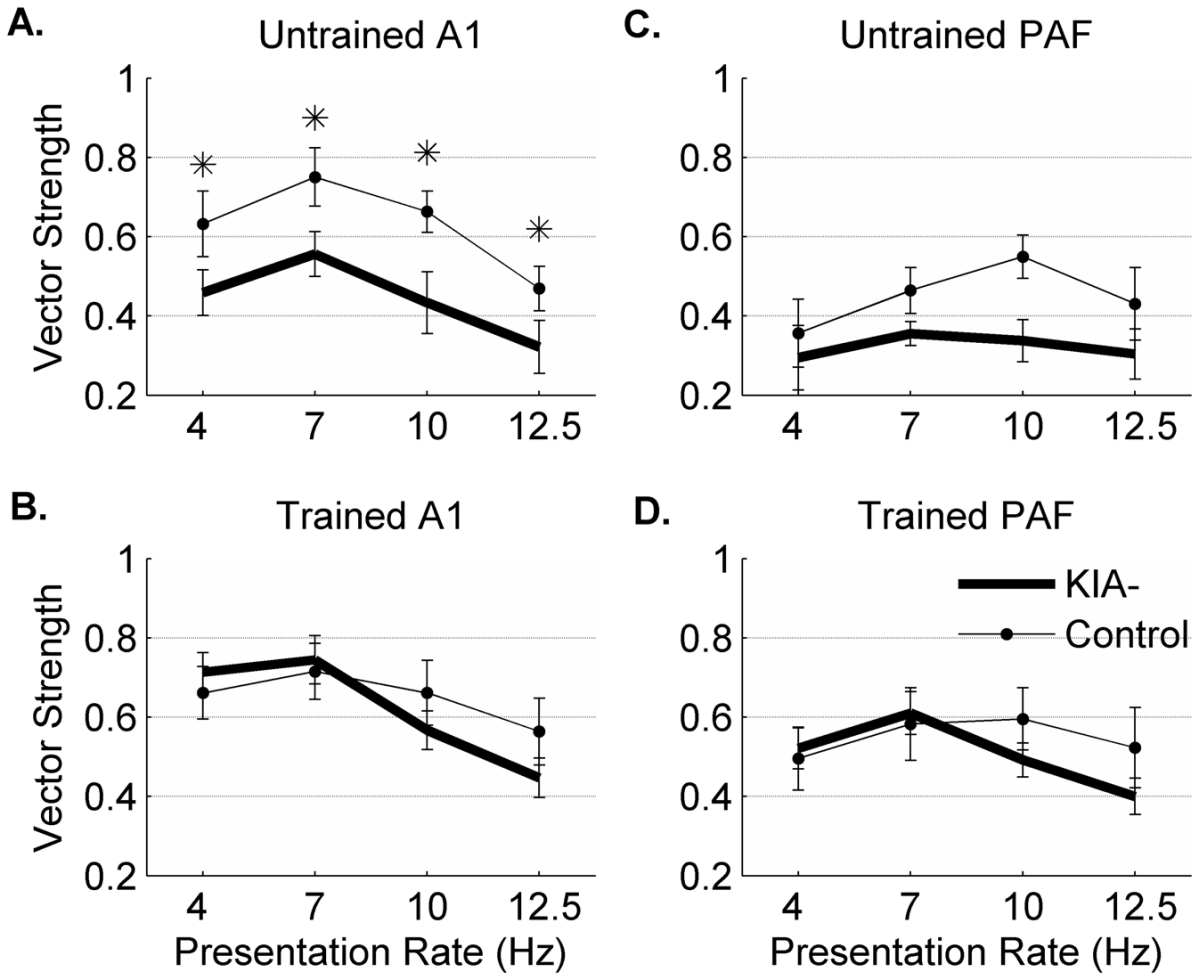
Training improves the ability of KIA- A1 and PAF sites to fire reliably to repetitive stimuli. **A**. Untrained KIA- A1 sites are significantly worse at following repetitive stimuli as measured by vector strength (* = p<0.01). **B**. Vector strength in control and KIA- A1 following auditory training. **C**. Vector strength in untrained control and KIA- PAF sites. **D**. Vector strength in control and KIA- PAF following auditory training.

Auditory cortex in untrained KIA- rats had significantly lower vector strength (VS) than control rats (Figure 7A) [31]. After auditory training, VS in KIA- A1 was no longer significantly different from control rats at any speed we tested (1-way ANOVA, F(1,6) = 0.18, p = 0.68; Figure 7A&C). There were no significant differences in VS in PAF across control and KIA- groups (4 Hz p = 0.67, 7 Hz p = 0.24, 10 Hz p = 0.06, 12.5 Hz p = 0.39). Training did significantly improve VS in KIA- PAF (0.32±0.1 in untrained KIA- PAF vs. 0.51±0.1 in trained KIA- PAF; 1-way ANOVA, F (1,6) = 16.1, p<0.01; Figure 7C&D), but did not affect VS in control PAF (0.45±0.1 in untrained control PAF vs. 0.55±0.1 in trained PAF; 1-way ANOVA, F(1,6) = 4.52, p = 0.08; Figure 7C&D). These results suggest that extensive speech training can improve neural firing to non-speech stimuli, which is consistent with recordings from dyslexic children before and after training [35]–[37], [39], [77,77].

### Training-induced plasticity improves local field potentials as well as action potential patterns

**Figure 8:**
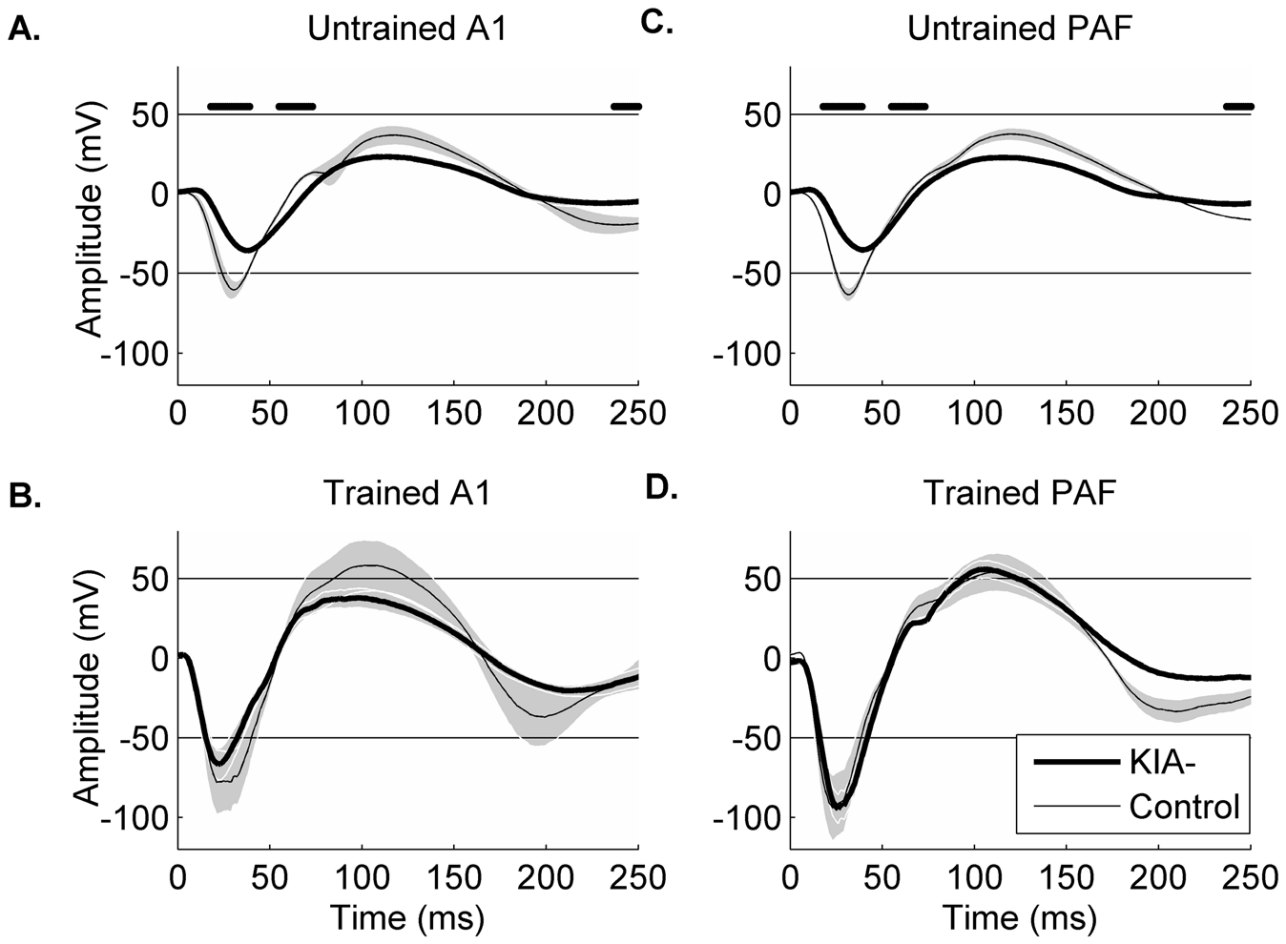
Extensive behavioral training shortens latency and increases amplitude of N1 component of LFP response. Responses are plotted with gray markers at −50 and 50 mV to help visualize differences across plots. Significant differences are marked by a black line. **A**. LFP response to the sound /dad/ in untrained control and KIA- A1. **B**. Extensive training improves onset latency and amplitude of the LFP response in KIA- A1. **C**. LFP response to the sound /dad/ in untrained control and KIA- PAF sites. As was seen in A1 recordings, latency and amplitude of KIA- PAF responses were significantly different from control recordings. **D**. Following training, there were not significant differences in the LFP response to the sound /dad/ between control PAF and KIA- PAF.

Since earlier studies of plasticity after dyslexia therapy used EEG or fMRI, we hypothesized that the neural plasticity we observed in the action potential patterns of multi-unit recordings of rats would also be visible in the local field potentials (LFPs). After speech discrimination training, LFPs in control A1 responded faster to the onset of the speech sound /dad/ (p<0.01; Figure 8A). Trained KIA- A1 LFPs also responded faster compared to untrained recordings (p<0.01; Figure 8B). N1 amplitude was significantly increased as a result of training in control A1 (−72.4±2.0 mV in untrained control A1 vs.−82.8.3±20.3 mV after training; p<0.01; Figure 8A) and in KIA- A1 (−41.3±1.5 mV in untrained KIA- A1 vs. −69.1±10.1 mV after training; p<0.01; Figure 8B). Latency of the N1 in control PAF was not significantly affected by training (p = 0.07; Figure 8C). The LFP in KIA- PAF had a longer latency following training (20.8±14.5 ms in untrained KIA- PAF vs. 42.0±0.9 ms after training; p<0.01; Figure 8D). After training, there was a significant increase in N1 amplitude in KIA- PAF (−18.9±3.5 mV in untrained KIA- PAF vs. −73.8±5.1 mV after training; p<0.01; Figure 8D). N1 amplitude in control PAF was also significantly increased by training (−42.1±5.2 mV in untrained control PAF vs. −94.6±10.4 mV after training; p<0.01; Figure 8C). Our observation that training induced plasticity improved neural discrimination performance of KIA- A1 and PAF sites suggests a possible neural basis for the success of current therapeutic options for humans with dyslexia.

## Discussion

### Summary of results

The behavioral data we collected confirmed our hypothesis that assumed *in utero* RNAi of the candidate-dyslexia gene *Kiaa0319* in rats would cause impaired speech sound discrimination in quiet and in noise. KIA- rats were significantly impaired at discriminating a target speech sound from distractor speech sounds in a variety of contexts and required twice the training time to perform at control levels. Training with truncated speech sounds led to improved performance on tasks using full length sounds in quiet and noisy conditions. KIA- rats who trained on truncated speech discrimination were more accurate on the other speech tasks compared to KIA- rats who had not trained on truncated speech. The neurophysiology data we collected demonstrates that behavioral training improved neural discrimination of consonants and reduced the neural variability in KIA- rats. Improved neural processing generalized to sounds that were not trained. These results provide a potential neural justification for the widespread use of intensive auditory training for dyslexia.

### Biological basis of dyslexia

People with dyslexia have impairments in phoneme identification and manipulation that are correlated with abnormal neural responses. Dyslexics have reduced cortical and thalamic responses to non-speech sounds and speech sounds in passive and active listening conditions [21], [78]–[80]. Auditory responses in the brainstem have similar amplitude in children with dyslexia as in normally developing children, but the trial-by-trial variability is significantly elevated [81]. Although the high heritability of dyslexia has been known for decades [14], [77], [82], [83], the specific genes responsible have become clear only recently [11], [12], [22], [84], [85]. A growing body of evidence from human and animal studies will establish which of these genes are responsible for the well documented impaired auditory processing impairments in dyslexia.

Variants in *KIAA031*9 have been associated with dyslexia in at least nine independent studies [11], [11], [12], [18], [19], [22], [86], [87], [87]–[90]. None of the known human mutations in *KIAA0319* eliminate the gene, but they do reduce expression of the protein [22], [90], [91].The best characterized mutation causes reduced expression due to transcriptional suppression by OCT-1 [92]. Future studies should include testing of rats with full knockout of *Kiaa0319* to test the consequences of complete protein elimination, However, the rat model we describe here mimics the functional knockdown seen in human cells by suppressing Kiaa0319 protein expression in the developing brain using RNAi and is likely a more accurate model of the functional consequence of *KIAA0319* variants in humans [91].

Cortical neurons affected by *in utero* RNAi of *Kiaa0319* have significantly increased membrane resistance and are therefore hyperexcitable compared to neurons transfected with control or rescue sequences [31]. This increased excitability appears to be responsible for the increased trial-by-trial variability seen in the neural responses of KIA- rats. Variability can negatively impact the brain's ability to reliably locate a stimulus onset and determine the identity of sounds [93], [94]. The increased variability in KIA- auditory cortex significantly impaired the ability of a classifier to use neural activity to identify various speech sounds [31]. These data suggested that decreased neural precision may be responsible for the impaired phonological awareness deficits in dyslexia. In the current study, we confirm that impaired neural encoding of speech sounds caused by *Kiaa0319* RNAi leads to impaired behavioral discrimination of speech sound stimuli. In addition to the 1–2% of neurons that were affected by the transfection, other studies have shown the Kiaa0319 targeting shRNA also alters non-transfected cells in cortex [16], [46], [95], [96]. For example, non-transfected GABAergic neurons have been seen in heterotopias along with transfected cells [46], [95], which suggests that more neurons in primary auditory cortex were likely affected in our study than could be visualized. The observed correlation between transfection rate and behavior suggests that the extent of the changes in non-tranfected cells is likely proportional to the number of transfected cells.

### Efficacy of RNAi in the current study

In the current study, we used RNA interference, which is a common technique for knocking down protein expression in the brain [16], [20], [31], [43], [45], [95], [97]. Though this technique has been used and proven to be effective in countless studies with immunohistochemistry [98]–[100], the techniques for demonstrating effective knockdown of the Kiaa0319 protein are not yet fully developed. The success of the Kiaa0319 specific shRNA used in the RNAi procedure has been shown in culture (Figure S1). Although at least one western blot for Kiaa0319 protein has been published [46], the current antibodies available for marking this protein are not as precise as many other antibodies on the market and therefore this western does not definitively prove the efficacy of the technique used in the current study. Therefore, it is likely (but not certain) that the RNAi procedure used in this study is successful at reducing the expression of Kiaa0319 protein. Definitive confirmation of this conjecture will depend on the development of an effective antibody for Kiaa0319 protein.

### A neural mechanism for training-induced plasticity

Many interventions for dyslexia focus on auditory processing to improve the mapping from phonemes to graphemes [35]–[38], [101]. These interventions for dyslexic children can reduce the variability in speech-evoked neural responses across repeats of each stimulus [35]–[38], [77]. The increase in firing amplitude seen after training could be due to a decrease in variability of onset latency, even if the response strength remains unchanged. Earlier studies hypothesized that the neural changes caused by training are due to a decrease in trial-by-trial variability [102].

Training could improve variability and thus improve speech sound processing through synaptic plasticity mechanisms that alter excitation and/or inhibition. Suppression of candidate-dyslexia genes affects the development of GABAergic neurons in the developing brain [97]. Normally, GABA regulates the balance between excitation and inhibition. In the dyslexic brain, improperly functioning GABA cells may contribute to the high excitability and variability of cortical neurons reported in our previous study [103]. Extensive auditory training, like the kind used in the current study, may cause LTP of GABAergic projections to cells affected by RNAi [104], which would reduce spontaneous firing and improve efficiency of downstream neurons [105], [106]. We hypothesize that extensive behavioral training using complex auditory stimuli, such as speech, will reduce membrane resistance in cortical neurons and additional studies are needed to test this hypothesis.

### Effect of Training on Neural Responses

There is a considerable amount of debate in the literature regarding the effect of behavioral training on neural responses to auditory stimuli. In the current study, we show that KIA- auditory cortex firing properties do change as a result of behavioral training, while control responses remain stable. Several recent studies have looked at the neural responses to auditory stimuli at various points throughout training. These studies used terminal acute recording techniques and therefore required a separate experimental group for each time point. Nevertheless, these studies clearly document the process of neural responses as they become better predictors of stimulus identity during the course of training [101], [102]. Chronic recordings of non-primate animals learning motor tasks show a progression of neural plasticity throughout training [103], [104]. Training studies using auditory stimuli would benefit from the use of chronic electrode implants to document the progress of neural plasticity in individual animals.

### Other candidate-dyslexia genes

It is possible that candidate-dyslexia genes could interfere with reading without affecting phoneme processing by interfering with cognitive processes such as working memory or visual attention. It is interesting to note that all four of the best characterized candidate-dyslexia genes (KIAA0319, *ROBO1, DYX1C1,* and *DCDC2*) interfere with auditory processing [12], [15], [19], [107]. Although the sequences of these genes are not related, each of these genes is required for normal neural migration [108]–[110]. The methods used in the current study could be used to evaluate the effect of other candidate-dyslexia genes on speech processing in rats. If future studies confirm that other genes can cause similar speech processing impairments to those described here, it will suggest that there is a common pathway to dyslexia through a variety of possible genetic mutations.

It is perhaps surprising that we can study dyslexia related genes in rats, since speech sounds have no ecological meaning to rats and rats cannot read. However, rats able to discriminate speech sounds in degraded conditions with thresholds that are indistinguishable from human participants [47], [48], [52], [53], [55]. The most likely explanation is that many of the basic auditory processing mechanisms used by mammals contribute to human speech processing. Our results suggest that assumed reduced expression of *KIAA0319* can cause dyslexia by increasing trial-by-trial variability in auditory cortex, which could impair phoneme processing and make reading more difficult because the mapping from phonemes to graphemes is compromised [81], [102], [111]. In addition, it is likely that trial-by-trial variability exists throughout the auditory system, and possibly even in other sensory systems. The observation that the auditory processing impairments in dyslexia are not limited to speech sounds confirms that the neural basis of dyslexia extends well beyond language-specific brain regions and is thus suitable for study in animal models [112]–[114]. Our observation that extensive training can significantly reduce trial-by-trial neural firing variability in our animal model suggests a possible means by which behavioral interventions could successfully treat dyslexia. Animal studies could be used to better understand how different forms of sensory and behavioral interventions impact phoneme processing. Given the substantial genetic and experiential heterogeneity among individuals with dyslexia, a simplified experimental model of the disorder is likely to prove valuable for comparing the neural and behavioral impacts of various interventions.

## Supporting Information

Figure S1 In cell western assay confirming effectiveness of Kiaa0319 shRNA against rat Kiaa0319. Columns 1–4 and rows A–C show culture wells containing transfected and processed Hek293 cells in triplicate (A–C, rows) transfected with four different conditions and detected with antibodies against an mRFP epitope tag. Column 1 wells were not transfected and this is the background staining level. Column 2 cultures were transfected with pCAG-Kiaa0319-mRFP and a mutant control shRNA that does not match Kiaa0319 coding sequence. The bright red in column 2 indicates intense expression above background of Kiaa0319-mRFP. Column 3 is the same pCAG-Kiaa0319-mRFP construct transfected in 2 with the addition of the shRNA used to knockdown Kiaa0319 in this study. Column 4 is a similar co transfection with another shRNA vector based on the mir-30 system that contains the same shRNA targeting sequence as the shRNA shown in column 3 experiments.
(TIFF)

Figure S2 *Rats with in utero* RNAi *of Kiaa0319 are able to learn a simple lever pressing task*. All rats (16 KIA- rats and 10 control rats) were first trained to press a lever, which triggered the presentation of the target sound (/dad/) and a sugar pellet reward. KIA- rats learned this task in the same amount of time as control rats (to criterion of 2 sessions of 100 presses; 113.4±14.2 minutes for KIA- rats vs. 141.5±27.2 minutes for controls; unpaired t-test, p = 0.30). After learning to press the lever, rats were transitioned to detection in which they were required to press the lever only when the target sound /dad/ was presented. Rats with *in utero* RNAi of *Kiaa0319* were not impaired in their ability to switch from free pressing to waiting for the target sound. Both groups were able to reach the performance criterion (10 sessions with a d'≥1.5) in approximately 5 days (Controls: 8.4±0.3 days vs. KIA-: 9.6±0.6 days; p = 0.17; Figure S1A). KIA- rats were not slower to respond to speech sounds compared to controls (Figure S2B). Responses by KIA- rats were faster on the first day of training, but were not different on any other training day. KIA- rats did not false alarm to silent catch trials more than control rats at any point during detection training (Figure S2C). Early in training KIA- rats missed more target sounds than controls, but were not significantly different from controls throughout the remainder of detection training (days 2 and 3 of detection training, one tailed t-test, p<0.01; Figure S2C).These observations indicate that i*n utero* RNAi of *Kiaa0319* does not significantly impair gross motor, sensory or cognitive abilities, which is consistent with earlier reports that KIA- rats can hear and have normal working memory. **A**. Both KIA- and control rats were able to learn a simple speech detection task within 7 days of training (14 sessions). **B**. KIA- rats responded as quickly as control rats except on the first day of training, when they were significantly faster (p<0.01). **C**. KIA- rats (thick black line) responded to the target sound (squares) less often than control rats (thin black line) during the second and third days of training (* = p<0.01). The false alarm rate was not significantly different between the two groups (triangles).
(TIF)

Figure S3 Figure For all behavioral training, we used a set of consonant-vowel-consonant (CVC) speech sounds that have been used in many previous studies. All speech sounds were recorded in our lab by a female, native English speaker and were shifted up by an octave to better accommodate the rat hearing range (Kawahara 1997). For the truncated speech task, we used only the first 40 ms of the speech sounds, shown in the bottom panel. This figure was reprinted with permission from Porter et al., 2011.
(TIF)

Figure S4 *Training affects basic neural firing properties to tonal stimuli in KIA- and Control rats.* We trained rats for 4 months on a variety of speech discrimination tasks (Figures 2&4, Main Text) and evaluated the effect of such training on neural firing properties. Training reduced onset latency in both KIA- A1 (25.8±0.6 ms in untrained vs. 17.7±0.7 ms after training; p<0.01) and PAF (45.6±7.1 ms in untrained vs. 29.5±2.2 ms after training, p = 0.01; Figure S4A). KIA- A1 neurons fired fewer evoked spikes after training (2.9±0.1 spikes in untrained vs. 2.4±0.1 spikes after training, p<0.01; Figure S4D). This reduction in action potentials may be related to the decrease in neural variability we observed (Figure 5, Main Text). Training induced shorter latencies (22.3±0.7 ms in untrained vs. 17.2±0.6 spikes after training, p<0.01; Figure S4A), narrower bandwidths (2.3±0.1 octaves in untrained vs. 1.9±0.1 octaves after training, p<0.01; Figure S4B), and had a greater number of driven action potentials to tones (2.8±0.1 spikes in untrained vs. 3.2±0.1 spikes after training, p<0.01; Figure S4D) in control rats (with scrambled shRNA). Thresholds were not affected by training in any group (Figure S4C). Following training, auditory cortex in KIA- and control rats were no longer significantly different in onset latency (A1 and PAF), bandwidth (A1 and PAF), and threshold (PAF). **A**. Training significantly shortened the onset latency in Control and KIA- A1 and KIA- PAF. No significant differences were seen in control PAF sites. **B**. Extensive behavioral training shortened bandwidths in Control A1 (2.3±0.1 octaves in untrained vs. 1.9±0.1 octaves after training, p<0.01), but had no effect on bandwidths in the other fields. **C**. Extensive behavioral training had no effect on auditory thresholds in any group or field (control A1; p = 0.72, KIA- A1; p = 0.06, control PAF; p = 0.90, KIA- PAF; p = 0.53). **D**. Extensive behavioral training increased the number of tone-evoked action potentials fired in control A1 (2.8±0.1 spikes in untrained vs. 3.2±0.1 spikes after training, p<0.01), but reduced the number of tone-evoked spikes fired in KIA- A1 (2.9±0.1 spikes in untrained vs. 2.4±0.1 spikes after training, p<0.01).
(TIF)

Figure S5 *Extensive behavioral training improves neural encoding of vowel sounds in control and KIA- auditory cortex.* After training, trial-by-trial variability in onset latency across sites in KIA- A1 and PAF as well as control PAF were significantly reduced (Figure 5, Main Text). Responses to consonant speech sounds were significantly more precise following training and were better able to encode the differences between consonant sounds (Figure 6, Main Text). We saw a similar effect in the encoding of vowel sounds following training. Vowel sounds are encoded using spike count over a single 400 ms analysis window. As reported previously, untrained control and KIA- A1 responded to vowel sounds with a high degree of variability, and these two sites performed worse at the vowel task than at the consonant task (Figure S5 A&B; data originally collected for and reported in). Untrained PAF in control animals was slightly worse at the vowel task than A1 in each group. Average performance by untrained control PAF sites was 64.4±2.4% correct vs. 73.4±0.6% correct in untrained control A1 (p<0.01; Figure S5C). Performance in untrained KIA- PAF sites was not significantly worse than untrained KIA- A1 sites (64.3±1.4% correct in PAF vs. 62.9±0.1% correct in A1; p = 0.71; Figure S5D). Following training, we noticed a slight (but not significant) improvement in the neural encoding of vowels. Trial-by-trial variability was reduced in every field (Figure S5 E–H and Figure 5, Main Text), which slightly improved the ability of each site to encode differences in vowel sounds. This result suggests that the specific training tasks we used benefitted consonant processing more effectively than vowel processing. **A**. A representative site from untrained control A1. The number of spikes encoded in response to each vowel sound was used to predict which sound evoked each single trial response. Data originally collected for and reported in Centanni et al. 2013. **B**. A representative site from untrained KIA- A1. The variability in neural firing was significantly higher in KIA- sites, which significantly impaired the ability of these sites to perform the vowel discrimination task. Data originally collected for and reported in Centanni et al., 2013. **C**. A representative site from untrained control PAF. **D**. A representative site from untrained KIA- PAF. **E**. A representative site from trained control A1. Though training did not have a significant impact on the classifier performance, the reduced variability in this field following training did provide some improvement on neural processing of vowels in this field. **F**. A representative site from trained KIA- A1. The improved variability in KIA- neurons after training did improve classifier performance on the vowel tasks, though this improvement was not significant. **G**. A representative site from trained control PAF. There was significant reduction in trial-by-trial variability in this field after training, and there was slight (but not significant) improvement in this fields' vowel classifier performance. **H**. A representative site from trained KIA- PAF. There was significant reduction in trial-by-trial variability in this field after training, and there was slight (but not significant) improvement in this fields' vowel classifier performance.
(TIF)

Figure S6 *An additional 4 weeks of behavior training causes additional plasticity in control rats.* The 4 weeks of additional training (as shown in Figure 4, Main Text) was also able to further reduce the trial-by-trial onset latency variability in control rats, but not KIA- rats as compared to group 1. In control A1, neural recordings from group 2 rats had lower trial-by-trial variability compared to group 1 (34.6±3.3 ms^2^ in group 1 vs. 20.3±3.2 ms^2^ in group 2; unpaired t-test, p = 0.01; Figure S6A and Figure 6, Main Text). Control PAF in group 2 was also less variable trial-by-trial as a result of the additional training (59.4±4.3 ms^2^ in group 1 vs. 29.5±2.6 ms^2^ in group 2; unpaired t-test, p<0.01; Figure S6A). Trial-by-trial variability in KIA- rats did not decrease with additional training (A1: 27.3±4.6 ms2 in group 1 vs. 29.4±4.5 ms2 in group 2; p = 0.72, PAF: 44.5±3.6 ms2 in group 1 vs. 44.5±3.9 ms2 in group 2, p = 0.99; Figure S6A). We observed an increase in neural discrimination (as measured by the nearest-neighbor classifier) ability selectively in control PAF. Neural activity from group 2 control PAF sites were better able to discriminate between pairs of consonants than group 1 control PAF (65.3±2.1% correct by group 1 vs. 77.2±5.5% correct by group 2; unpaired t-test, p<0.01; Figure S6B). Control and KIA- A1 and KIA- PAF sites did not improve on the neural consonant discrimination task as a result of additional training (unpaired t-tests; p = 0.29, p = 0.16, and p = 0.88, respectively; Figure S6B). Similarly, no group experienced an increase in neural vowel discrimination performance as a benefit of additional training (Control A1, p = 0.05; Control PAF, p = 0.36; KIA- A1, p = 0.42; KIA- PAF, p = 0.70; Figure S6C). The result that additional training did not provide additional neural plasticity in KIA- rats suggests that there may be a limit in how beneficial behavioral therapy can be in mediating the impairment caused by variants in *Kiaa0319*. **A**. The additional training received by group 2 caused a significant reduction in trial-by-trial variability in control A1 (p = 0.01) and control PAF (p<0.01). No significant changes were seen in either field in KIA- rats (p = 0.72 and p = 0.99 in A1 and PAF respectively). **B**. Additional training improved the ability of control PAF sites to perform the consonant neural discrimination task (p<0.01), but this training did not improve classifier performance in control A1 (p = 0.29), KIA- A1 (p = 0.16), or KIA- PAF (p = 0.88). **C**. Additional training did not improve the ability of neural activity in any group or field to perform the neural discrimination task using vowel stimuli. Control A1; p = 0.05, KIA- A1; p = 0.42, control PAF; p = 0.36, KIA- PAF, p = 0.70.
(TIF)
