# Supplementary figures and images for: Speech Sound Processing Deficits and Training-Induced Neural Plasticity in Rats with Dyslexia Gene Knockdown
Source: PLoS One. 2014 May 28;9(5):e98439. doi: 10.1371/journal.pone.0098439 (PMC4037188; doi:10.1371/journal.pone.0098439)

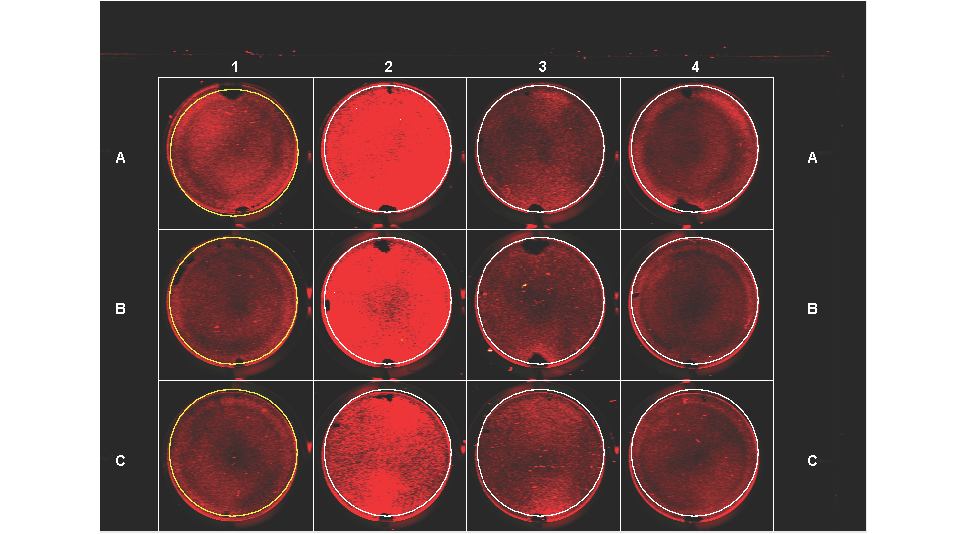

Supplement: Figure S1 — In cell western assay confirming effectiveness of Kiaa0319 shRNA against rat Kiaa0319. Columns 1–4 and rows A–C show culture wells containing transfected and processed Hek293 cells in triplicate (A–C, rows) transfected with four different conditions and detected with antibodies against an mRFP epitope tag. Column 1 wells were not transfected and this is the background staining level. Column 2 cultures were transfected with pCAG-Kiaa0319-mRFP and a mutant control shRNA that does not match Kiaa0319 coding sequence. The bright red in column 2 indicates intense expression above background of Kiaa0319-mRFP. Column 3 is the same pCAG-Kiaa0319-mRFP construct transfected in 2 with the addition of the shRNA used to knockdown Kiaa0319 in this study. Column 4 is a similar co transfection with another shRNA vector based on the mir-30 system that contains the same shRNA targeting sequence as the shRNA shown in column 3 experiments. (TIFF) [file pone.0098439.s001.tiff]

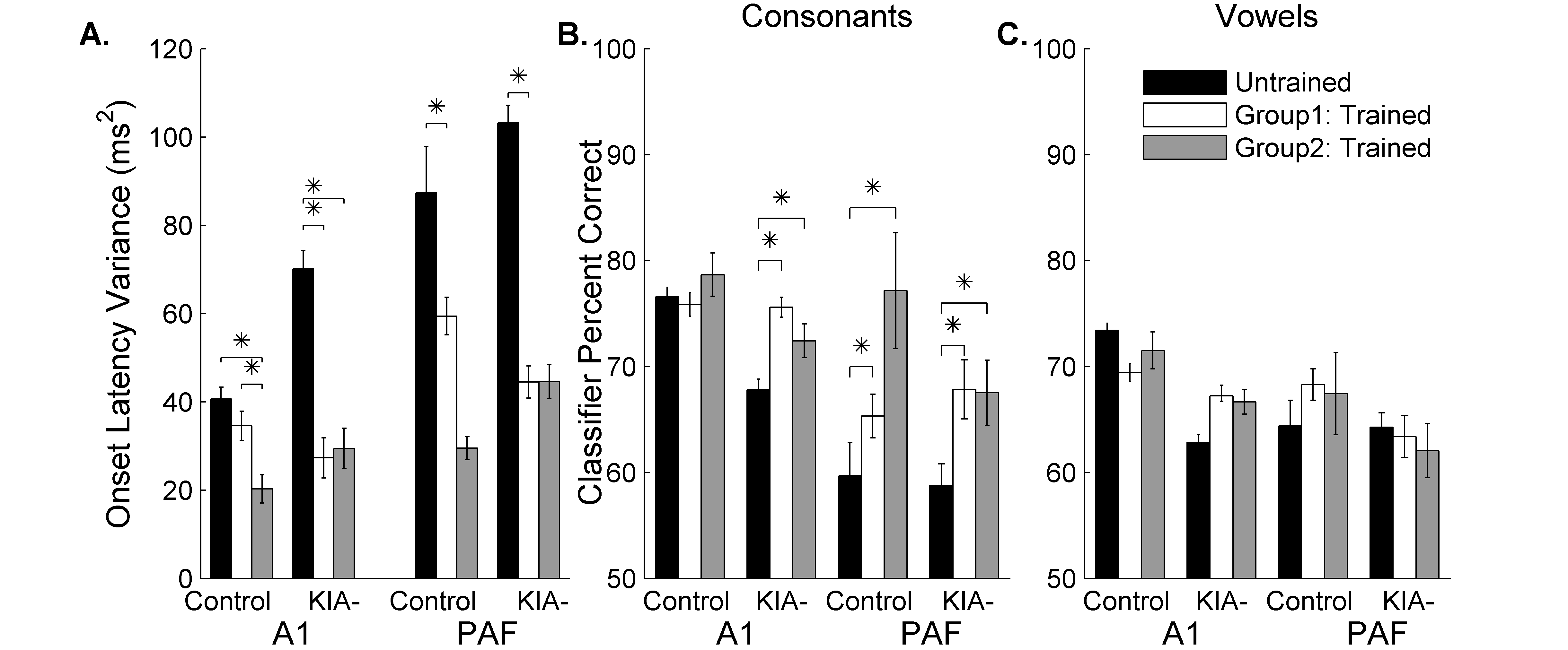

Supplement: Figure S6 — An additional 4 weeks of behavior training causes additional plasticity in control rats. The 4 weeks of additional training (as shown in Figure 4, Main Text) was also able to further reduce the trial-by-trial onset latency variability in control rats, but not KIA- rats as compared to group 1. In control A1, neural recordings from group 2 rats had lower trial-by-trial variability compared to group 1 (34.6±3.3 ms2 in group 1 vs. 20.3±3.2 ms2 in group 2; unpaired t-test, p = 0.01; Figure S6A and Figure 6, Main Text). Control PAF in group 2 was also less variable trial-by-trial as a result of the additional training (59.4±4.3 ms2 in group 1 vs. 29.5±2.6 ms2 in group 2; unpaired t-test, p<0.01; Figure S6A). Trial-by-trial variability in KIA- rats did not decrease with additional training (A1: 27.3±4.6 ms2 in group 1 vs. 29.4±4.5 ms2 in group 2; p = 0.72, PAF: 44.5±3.6 ms2 in group 1 vs. 44.5±3.9 ms2 in group 2, p = 0.99; Figure S6A). We observed an increase in neural discrimination (as measured by the nearest-neighbor classifier) ability selectively in control PAF. Neural activity from group 2 control PAF sites were better able to discriminate between pairs of consonants than group 1 control PAF (65.3±2.1% correct by group 1 vs. 77.2±5.5% correct by group 2; unpaired t-test, p<0.01; Figure S6B). Control and KIA- A1 and KIA- PAF sites did not improve on the neural consonant discrimination task as a result of additional training (unpaired t-tests; p = 0.29, p = 0.16, and p = 0.88, respectively; Figure S6B). Similarly, no group experienced an increase in neural vowel discrimination performance as a benefit of additional training (Control A1, p = 0.05; Control PAF, p = 0.36; KIA- A1, p = 0.42; KIA- PAF, p = 0.70; Figure S6C). The result that additional training did not provide additional neural plasticity in KIA- rats suggests that there may be a limit in how beneficial behavioral therapy can be in mediating the impairment caused by variants in Kiaa0319. A. The additional tr [file pone.0098439.s006.tif]
